# Dosimetric comparison of MR-linac-based IMRT and conventional VMAT treatment plans for prostate cancer

**DOI:** 10.1186/s13014-021-01858-7

**Published:** 2021-07-21

**Authors:** Vanessa Da Silva Mendes, Lukas Nierer, Minglun Li, Stefanie Corradini, Michael Reiner, Florian Kamp, Maximilian Niyazi, Christopher Kurz, Guillaume Landry, Claus Belka

**Affiliations:** 1 Department of Radiation Oncology, University Hospital, LMU Munich, Marchioninistraße 15, 81377 Munich, Germany; 2 German Cancer Consortium (DKTK), Partner Site Munich, Munich, Germany; 3 Department of Radiation Oncology, Cologne University Hospital, Cologne, Germany

**Keywords:** IMRT, VMAT, IGRT, MR-guidance, MRIdian linac, Low-field MR-linac, Prostate

## Abstract

**Background:**

The aim of this study was to evaluate and compare the performance of intensity modulated radiation therapy (IMRT) plans, planned for low-field strength magnetic resonance (MR) guided linear accelerator (linac) delivery (labelled IMRT MRL plans), and clinical conventional volumetric modulated arc therapy (VMAT) plans, for the treatment of prostate cancer (PCa). Both plans used the original planning target volume (PTV) margins. Additionally, the potential dosimetric benefits of MR-guidance were estimated, by creating IMRT MRL plans using smaller PTV margins.

**Materials and methods:**

20 PCa patients previously treated with conventional VMAT were considered. For each patient, two different IMRT MRL plans using the low-field MR-linac treatment planning system were created: one with original (orig.) PTV margins and the other with reduced (red.) PTV margins. Dose indices related to target coverage, as well as dose-volume histogram (DVH) parameters for the target and organs at risk (OAR) were compared. Additionally, the estimated treatment delivery times and the number of monitor units (MU) of each plan were evaluated.

**Results:**

The dose distribution in the high dose region and the target volume DVH parameters (D_98%_, D_50%_, D_2%_ and V_95%_) were similar for all three types of treatment plans, with deviations below 1% in most cases. Both IMRT MRL plans (orig. and red. PTV margins) showed similar homogeneity indices (HI), however worse values for the conformity index (CI) were also found when compared to VMAT. The IMRT MRL plans showed similar OAR sparing when the orig. PTV margins were used but a significantly better sparing was feasible when red. PTV margins were applied. Higher number of MU and longer predicted treatment delivery times were seen for both IMRT MRL plans.

**Conclusions:**

A comparable plan quality between VMAT and IMRT MRL plans was achieved, when applying the same PTV margin. However, online MR-guided adaptive radiotherapy allows for a reduction of PTV margins. With a red. PTV margin, better sparing of the surrounding tissues can be achieved, while maintaining adequate target coverage. Nonetheless, longer treatment delivery times, characteristic for the IMRT technique, have to be expected.

## Background

Prostate cancer (PCa) is the second most common solid tumour diagnosed in men of Western Europe and the United States [1]. External beam radiation therapy (EBRT) is one of the treatment options chosen for localised PCa. This technique has evolved substantially over the last decades, maximizing the dose of radiation delivered to the tumour, while decreasing normal tissue toxicity and sparing organs at risk (OAR). Technological improvements in planning, imaging and delivery of the treatment have led to the development of highly conformal techniques, such as intensity modulated radiation therapy (IMRT) and volumetric modulated arc therapy (VMAT) [2, 3].

Among the advantages reported for VMAT, in comparison to step-and-shoot IMRT, are shorter treatment delivery times, decreasing the influence of intra-fraction motion, the need of less monitor units (MU) and a potential better target coverage and dose conformity [4–6]. It is the technique of choice for the treatment of PCa in our clinic.

Inter- and intra-fractional anatomical variations must be considered when performing EBRT treatments [7, 8]. This explains the importance of image guided radiation therapy (IGRT): it allows for the adjustment of the patients’ daily setup and correction of target positioning during treatment delivery, helping enhancing the precision and accuracy of dose delivery [9–12]. Additionally, margins applied to the clinical target volume (CTV), accounting for internal organ motion, variability of the target position and organ deformation, can be reduced, and consequently, the surrounding healthy tissue can be better spared [11, 13]. For PCa, Deutschmann et al. stated that if inter- and intra-fractional variations during RT delivery are properly managed, treatment margin reduction to 3 mm might be reasonable [14].

IGRT strategies in current use are ultrasound (US), 2D X-ray imaging, magnetic resonance (MR) imaging or computed tomography (CT) [11, 15]. In our institution, PCa patients have been treated with VMAT since 2015 under cone beam computed tomography (CBCT)- and US-based IGRT. CBCT is one of the most common IGRT techniques for PCa RT treatments with conventional linear accelerators (linacs) [16]. This technique has, however, some limitations, such as its image quality (image blurring, artefacts and its poor soft tissue contrast) and the dose exposure to the patient [16, 17]. Advantages of US imaging are its real time imaging capability and a good soft tissue contrast, without delivering any further dose to the patient. As limitations, there is the inaccessibility of tissue shielded by bone or air, the susceptibility for imaging artefacts and the user dependency [18].

In recent years, both a low-field (0.35 T) MR-linac (ViewRay MRIdian, ViewRay Inc., Oakwood Village, OH, USA) and a high-field (1.5 T) MR-linac (Elekta Unity, Elekta AB, Stockholm, Sweden) were clinically introduced [19]. The low-field MR-linac, which is the system of interest for the current study, is a hybrid system, combining a conventional linac and an MR scanner for MR-guided radiotherapy (MRgRT), with the possibility of automated beam gating. A VMAT dose delivery technique is currently not available. Instead, a step-and-shoot IMRT (ssIMRT) dose delivery technique is used. This hybrid machine is able to acquire daily 3D volumetric MR images, to which a CT image is registered for enabling dose calculation of the anatomy of the day, as well as plan re-optimisation for online plan adaptation. MR images are characterised by an improved soft tissue contrast, which is very important for an accurate delineation of the target volume and surrounding organs. Moreover, with the low-field system 2D cine sagittal MR images can also be acquired during treatment for intra-fraction image guidance, allowing not only to monitor the target’s movement but also to deliver a gated treatment beam [20]. The information provided by MR-guidance is well suited for PCa, leading to better visualisation of the prostate and surrounding tissue than CT, thus giving an improved certainty of the position of the tumour [8, 21].

Several studies comparing the plan quality provided by IMRT and VMAT have been conducted through the years [4, 6, 22–34]. Studies comparing the treatment plan quality provided by VMAT and IMRT techniques for conventional linacs found VMAT to have better treatment efficiency and shorter treatment delivery times, as well as better dosimetric outcomes for the target and OARs [4–6, 24–26, 33]. More recently, studies describing MRgRT for lung, spine, prostate, rectum, pancreas, liver and kidney were performed, where the impact of reduced margins and plan adaptation showed that at comparable dose to the target a dose reduction to OARs was possible [8, 27–30, 32, 35–41]. Park et al. performed a treatment plan comparison for PCa between conventional linac-based VMAT plans and the MRIdian’s ^60^Co predecessor system (MR-^60^Co), where smaller planning target volume (PTV) margins for the MR-^60^Co -based IMRT technique were used [29]. The quality of MR-^60^Co-based IMRT plans was comparable to the quality presented by VMAT plans. Furthermore, lower doses to the rectum and the bladder were seen for MR-^60^Co IMRT plans. Other plan quality comparisons for low-field MR-linac-based IMRT systems were also performed for spine and lung [27–32]. Two other studies investigating and comparing the plan quality of high-field MR-linac-based IMRT plans and conventional linac-based VMAT plans for PCa were found [36, 39]. In both studies, identical PTV margins were used for both techniques, and a comparable plan quality was found between both techniques. At the time of writing, there was no study comparing treatment plan quality between low-field MR-linac IMRT (labelled IMRT MRL in this study) and conventional VMAT plans with identical margins, as well as with the reduced margin clinically used with MRgRT.

The aim of this study was to evaluate and compare the performance of IMRT MRL plans, planned for low-field MR-linac delivery, and clinical conventional linac-based VMAT plans, for the treatment of PCa. We evaluated the plan quality in terms of dose-volume histogram (DVH) parameters, as well as the conformity and homogeneity of the dose distribution. Furthermore, estimated treatment delivery times and number of MU were compared. Additionally, the potential benefit of MR-guidance was estimated, by using PTV margins adjusted for online adaptive MRgRT, as currently clinically adopted in our institution.

## Materials and methods

### Patient data and PTV margin concept

20 anonymized data sets of patients with localised PCa undergoing definitive radiotherapy (low and intermediate risk) [42] and treated at our institution from June 2017 to September 2019, were retrospectively selected for this planning study. Depending on the risk stratification, the prescribed dose was 74 Gy to the PTV in 37 fractions (2 Gy per fraction, daily) for 7 patients affected by low risk PCa, and 76 Gy to the PTV in 38 fractions (2 Gy per fraction, daily) for 13 patients affected by intermediate risk PCa.

Patients were scanned in the supine position using a CT scanner (Toshiba Aquilion LB, Canon Medical Systems, Japan). To provide geometric reproducibility, patients were instructed to have a comfortably filled bladder and an empty rectum. The selected patients were treated using a conventional Elekta Versa HD linear accelerator with an Agility multileaf collimator (MLC) consisting of 160 leaves, with a leaf-width of 5 mm. For image-guidance, two modalities were used: a daily CBCT and an US image. The CBCT was acquired in the beginning of each fraction to align the patient; the US images were captured in real-time during treatment in order to monitor the intra-fraction prostate motion, with the Elekta Clarity Autoscan system (Elekta AB, Stockholm, Sweden).

The CTV and the OARs were contoured on the planning CT data set (1 × 1 × 3 mm^3^) by an experienced radiation oncologist and according to the institutional protocol. The CTV delineation depended on the PCa risk presented by the patient: for the low risk group the CTV was only covering the prostate; for the intermediate risk group the proximal seminal vesicles were also included. The orig. PTV margins were defined as an isotropic expansion of 6 mm of the CTV in all directions, except posteriorly, where an expansion of 5 mm towards the rectum was used, following the institutional protocol for CBCT guided treatments. The delineation of the clinical PTV expansion as well as the OARs was done with a clinical treatment planning system (TPS) Oncentra MasterPlan Version 4.5 (Nucletron B.V., Veenendaal, Netherlands), which is used for delineation in our clinic.

For treatment plans currently delivered at the low-field MR-linac at our clinic, reduced margins adjusted for MRgRT are used [8, 28, 40, 43]. This reduction is supported by the additional information given by MR imaging with its better soft tissue contrast [39], the possibility to perform online adaptation (in case this is deemed necessary by the physician in charge) [40] as well as the delivery of a gated beam based on the 2D cine sagittal MR images acquired during irradiation [43]. This margin, designated in this study as red. PTV margin, was defined as an isotropic expansion of 4 mm of the CTV in all directions, except posteriorly, where an expansion of 3 mm towards the rectum was applied, similarly to what has been suggested and applied for prostate tumours at other institutions [8, 10, 40, 44]. In clinical practice for the MR-linac, delineation is done on an (1.5 × 1.5 × 1.5 mm^3^) MRI. In Viewray MRIdian TPS Version 5 (ViewRay Inc., Oakwood Village, OH, USA), contours are always defined as multiples of the resolution of the planning image, leading to an expansion of 4.5 mm of the CTV in all directions, instead of 4 mm, except posteriorly towards the rectum, where an expansion of 3 mm is obtained, as intended.

In this study we used (1 × 1 × 3 mm^3^) CT images in the low-field MR-linac TPS for planning. Thus the red. margin was, effectively, an expansion of 4 mm of the CTV in the left-right and anterior direction, while in the superior-inferior direction, as well as posteriorly towards the rectum, an expansion of 3 mm was obtained. For the orig. margin, with an expansion of 6 mm of the CTV in the superior-inferior direction, this feature of the TPS does not cause any changes on the effective margin.

### Planning technique

In this planning study, two sets of IMRT MRL plans were developed and compared to the already existing clinical VMAT plans. Table 1 shows the different specifications for both machines and TPSs investigated in this study. The IMRT MRL plans were generated using the same CT and delineations, except from also applying a reduced PTV margin, as used for the VMAT plans.

#### VMAT

VMAT plans were generated using a 6 MV photon beam, with the clinical Monaco Version 5.11.01 TPS (Elekta AB, Stockholm, Sweden) and the Agility MLC. For all patients included in this study a VMAT plan using two coplanar arcs was calculated using a Monte Carlo algorithm, with a calculation grid size of (0.3 cm)^3^ and a statistical uncertainty during dose prediction of 1%. These plans were delivered clinically.

#### IMRT MRL

IMRT MRL plans were created using Viewray MRIdian TPS Version 5 (ViewRay Inc., Oakwood Village, OH, USA). The low-field MR-linac system generates a 6 MV flattening-filter-free (FFF) photon beam in the presence of a 0.35 T magnetic field, using the RayZR MLC, a double-stacked and double-focused 138-leaf MLC with 4.15 mm effective leaf-width at isocentre. The dose calculation algorithm is also based on Monte Carlo simulation, with a calculation grid size of (0.3 cm)^3^ and a statistical uncertainty during dose prediction of 1%.

In order to mimic a VMAT dose distribution, while using the same isocentre as for the VMAT plans, 17 equally spaced beams, avoiding the couch’s top edges in order to avoid dosimetric uncertainty, were chosen to create the IMRT MRL plans.

Treatment plans using two different PTV margins were generated. In scenario 1, orig. PTV margins (used also in the clinical VMAT plans) were used for treatment planning, in order to allow the comparison of the performance of the two machines and TPSs, under similar conditions. In scenario 2, red. PTV margins were used, to estimate the potential DVH-based dosimetric benefits of MRgRT.

All IMRT MRL plans were generated by a single medical physicist. This was not the case for the VMAT plans due to their clinical nature. IMRT MRL plans were also evaluated by an experienced radiation oncologist.

**Table 1:** Machine specific parameters for both linacs and the TPSs parameters used for this study

Device specific parameters	Standard linac	Low-field MR-linac
SAD (cm)	100	90
Linac calibration	At the maximum dose point: 100 MU correspond to 1 Gy	
Effective leaf width (mm)	0.5	0.415
Field size at isocentre (cm^2^)	40 × 40	27.4 × 24.1
Multileaf collimator	Rounded leaf edges	double-stacked, double-focused leaves
IMRT technique	VMAT	ssIMRT
Photon energy (MV)	6	6
Flattening filter	FF	FFF
Static magnetic field (T)	–	0.35
TPS parameters:		
Calculation grid size (cm^3^)	0.3 × 0.3 × 0.3	0.3 × 0.3 × 0.3
Statistical uncertainty (%)	1	1
Dose calculation algorithm	XVMC	KMC

### Treatment planning objectives

The VMAT plans were not normalised as the final plans are a result of a constrained optimisation, for which at first the constraints for OARs are applied and after that step the algorithm tries to fulfil the target’s dose objective. For each patient, both IMRT MRL plans were normalised to the PTV D_95%_ achieved by the corresponding VMAT plan. In addition, the plans were generated such that the maximum dose would not exceed 107% of the prescribed dose. The following OARs were included in the analysis: rectum, bladder and femoral heads. Internal clinical guidelines, based on tolerance doses and volumes of the OARs suggested by QUANTEC [45], were followed and are presented in Table 2.

**Table. 2:** Dose-volume constraints used for OARs

OAR	Dose-volume constraint (% of total volume)
Rectum	V_70Gy_ < 15%
	V_60Gy_ < 25%
	V_50Gy_ < 50%
Bladder	V_70Gy_ < 20%
	V_60Gy_ < 30%
	V_50Gy_ < 50%
Femoral heads	V_50Gy_ < 10%

### Plan evaluation and comparison

VMAT dose cubes were imported into the low-field MR-linac TPS in order to analyse and compare all the plans using a single system. The PTV dose and coverage were assessed by the near maximum (D_2%_), the near minimum (D_98%_), the median (D_50%_), as well as the V_95%_ of the prescribed dose. The degree of homogeneity of the plans was measured by the homogeneity index (HI), which evaluates the homogeneity of the dose distribution within the PTV [46, 47]. The closer the HI is to zero the higher the homogeneity of the dose distribution will be [48]:

1 $${HI}=\frac{{{D}}_{2{\%}}-{{D}}_{98{\%}}}{{{D}}_{50{\%}}}.$$

The conformity index (CI) considers the shape of the target and reference isodose, as well as the degree of spatial intersection of the two volumes [49, 50]. When a reference isodose is surrounding the PTV completely, not extending to the surrounding tissue, CI equals 1, meaning a perfect hypothetical conformal treatment is achieved. A deviation from perfect conformity gives a lower score. The CI is calculated, according to Paddick for the 95%-isodose, which takes into account the target volume covered by 95% of the prescribed dose (${V}_{95\% PTV}$), the target volume (${V}_{PTV}$) and the total volume covered by 95% of the prescribed dose (${V}_{95\% isodose}$) [51]:

2 $${CI}=\frac{{\left({{V}}_{95{\%} {PTV}}\right)}^{2}}{{{V}}_{{PTV}}\times {{V}}_{95{\%} {isodose}}}.$$

According to our internal clinical guidelines, relevant dose-volume parameters for the OARs, such as V_70Gy_, V_60Gy_, V_50Gy_ for the rectum and bladder were reported and compared. For the femoral heads, since a dose of 50 Gy was never achieved, it was decided instead to analyse the near maximum, D_2%_. To evaluate the low dose exposure, total volumes V_40Gy_ and V_25Gy_ were chosen, as in a study by Hoffmann et al. [3]. We used the patients’ outer contour defined for the superior-inferior extent of the planning CT scans. The average treatment delivery time (from first beam-on to final beam-off, estimated by both TPS) for all three plan scenarios, as well as the number of MU required for each plan, were compared.

### Statistical analysis

The IBM SPSS Statistics Version 26.0 software (IBM Corporation, Armonk, New York) was used to perform Wilcoxon signed-rank sum tests to investigate differences for each single DVH parameter independently between VMAT and IMRT MRL plans. Differences were considered statistically significant for *p* value ≤ 0.05. No correction for multiple testing was done since the analysis was performed for each parameter individually, and comparisons between IMRT MRL plans with orig. and red. margins were not performed.

## Results

**Fig. 1:**
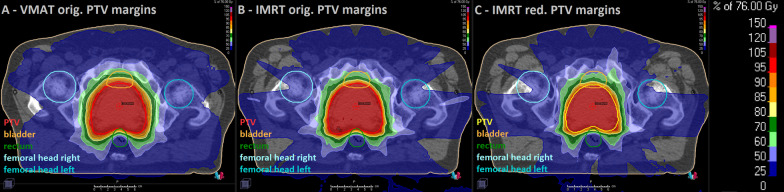
Dose distributions of the three different treatment plans for Patient 1, showing the PTV and the OARs. **A** VMAT plan with orig. PTV margins. **B** IMRT MRL plan with orig. PTV margins. **C** IMRT MRL plan with red. PTV margins

Clinically acceptable IMRT MRL plans, with orig. and red. PTV margins, were achieved for all patients. The mean original PTV volume was 184.6 ± 49.3 cc (range 97.4–319.1 cc), whilst the mean PTV volume with red. margins was 138.1 ± 40.4 cc (range 53.6–242.2 cc), resulting in a reduction on average of 25% of the original volume. Exemplary dose distributions of the three different treatment plans, for a prescription dose of 76 Gy, for Patient 1 are shown in Fig. 1. The VMAT and both IMRT MRL dose distributions are visually similar in the high dose region. The VMAT plan PTV dose-volume parameters D_98%_ and D_2%_ were 73.3 and 78.6 Gy, respectively. For the IMRT MRL plans, the respective values were 73.4 and 78.9 Gy for orig. PTV margins, and 73.2 and 79.3 Gy, for red. PTV margins. The largest differences were seen in the low dose bath. For VMAT/IMRT MRL with orig. PTV margins V_40Gy_ and V_25Gy_ were 695 cc and 1848 cc versus 634 cc and 1916 cc, respectively. As expected, the IMRT MRL with red. PTV margins plan allowed better OARs sparing and a further reduction of the low dose bath, with V_40Gy_ and V_25Gy_ of 528 cc and 1558 cc, respectively.

**Fig. 2:**
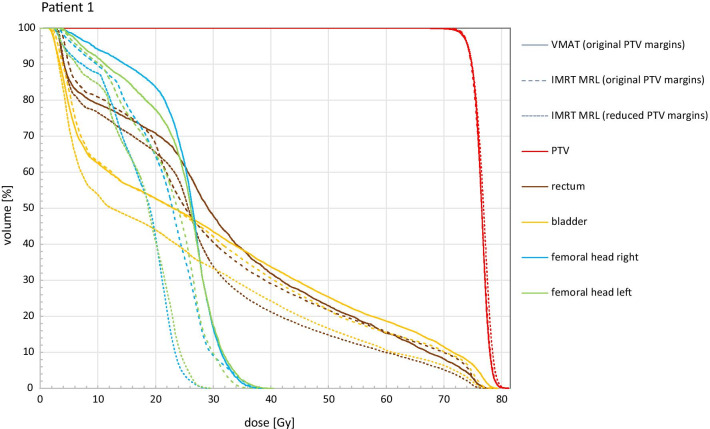
DVH curves for the three different treatment plans, for Patient 1. The orig. VMAT plan (solid lines), the IMRT MRL plan with orig. PTV margins (dashed lines) and the IMRT MRL plan with red. PTV margins (dotted lines) are shown. The target volumes (with different margins), the rectum, bladder and femoral heads’ volumes are plotted

The corresponding DVHs of the VMAT plan as well as both IMRT MRL plans are shown in Fig. 2. Both IMRT MRL plans were normalised to the PTV D_95%_, as in the VMAT plans. The treatment planning constraints for target and OARs were achieved in all plans: DVH curves for target volumes are almost overlapping, while OARs present similar DVH curves for both VMAT and IMRT MRL plans, when orig. PTV margins are applied. However, a reduction of the dose in all OARs for the IMRT MRL red. PTV margins plans was observed, as expected.

**Fig. 3:**
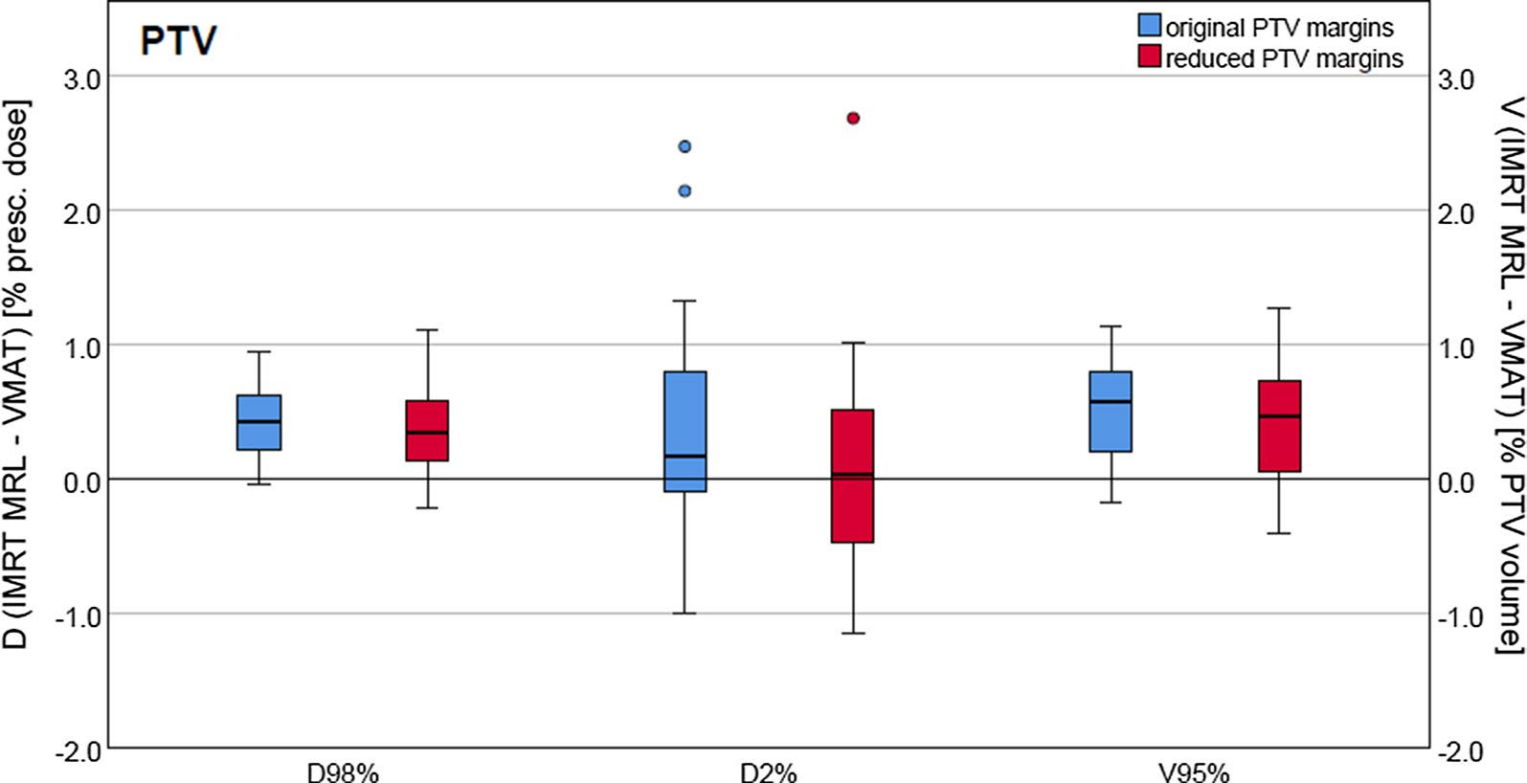
DVH parameter differences for the PTV for all patients plotted as boxplots. The D_98%_ and the D_2%_ differences are plotted along the left vertical axis, relative to the prescribed dose, and the V_95%_ differences are plotted along the right vertical axis, relative to the PTV volume. The results using orig. and red. PTV margins are plotted in blue and in red, respectively. The boxplots indicate the spread of the central 50% of the data, denominated as the interquartile range (IQR). The median, the 25th and the 75th percentiles are also shown. The upper and lower whiskers represent data outside the IQR but inside the range defined by 1.5 × IQR. Outliers are defined as values outside the whiskers’ range

Boxplots of the distribution of the differences of DVH parameters for the target volumes of all patients, between the IMRT MRL and the VMAT plans, for both orig. and red. PTV margins are presented in Fig. 3. The D_98%_, D_2%_ and V_95%_ achieved for the IMRT MRL plans for both PTV margins were on average slightly higher than the ones reported for the VMAT plans, with median differences below 0.6%. For a few cases the D_2%_ difference exceeded 2% for the IMRT MRL plans. However, the D_2%_ was still within the tolerance limits. The Wilcoxon matched-pair signed rank test showed there was no significant difference in D_2%_ between VMAT and IMRT MRL (*p* > 0.05), as opposed to D_98%_ and V_95 %_, where differences were statistically significant (*p* ≤ 1 × 10^− 5^). Deviations between IMRT MRL and VMAT were, however, below 1% in most cases.

**Fig. 4:**
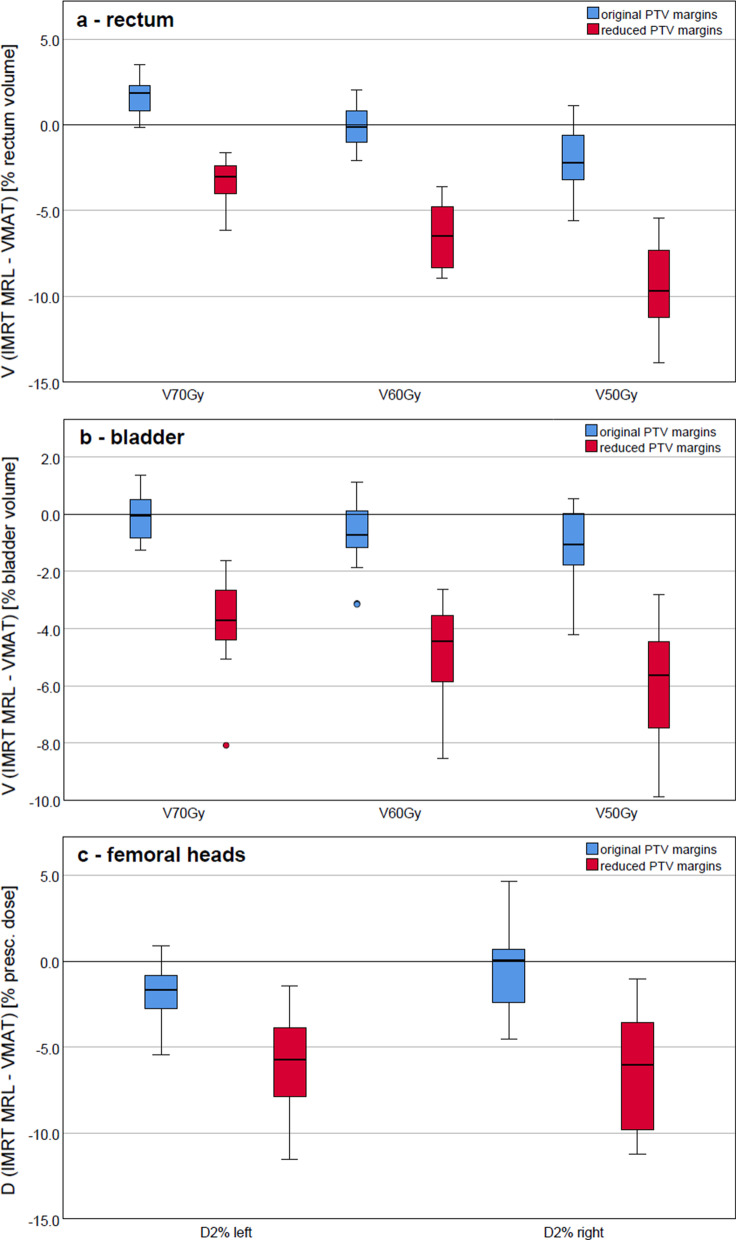
DVH parameter differences for the OARs, for all patients, as boxplots are shown. V_70Gy_ V_60Gy_, V_50Gy_ for **a** rectum and **b** bladder, relative to the rectum and bladder volume, respectively, and D_2%_ for **c** femoral heads, relative to the prescribed dose. The results using orig. and red. PTV margins are plotted in blue and in red, respectively. The boxplots indicate the spread of the central 50% of the data, denominated as the IQR (interquartile range). The median, the 25th and the 75th percentiles are also shown. The upper and lower whiskers represent data outside the IQR but inside the range defined by 1.5 × IQR. Outliers are defined as values outside the whiskers’ range

The distribution of the differences between dose-volume parameters of the OARs for all patients, between IMRT MRL and VMAT plans, orig. and red. PTV margins, is presented by the boxplots shown in Fig. 4. When using the orig. PTV margins, the V_70Gy_ of the rectum was on average 1.7% higher for the IMRT MRL plans than for the VMAT plans (*p* ≤ 1 × 10^− 5^). However, for IMRT MRL plans, the V_60Gy_ was roughly the same (16 ± 5)% as for VMAT plans (16 ± 4)%, with no significant difference. The V_50Gy_ was slightly lower for IMRT MRL (22 ± 6)% than for VMAT plans (24 ± 6)%, but statistically significant (*p* ≤ 1 × 10^5^). For red. PTV margins the V_70Gy_, V_60Gy_ and V_50Gy_ were always smaller for IMRT MRL plans, which was statistically significant (*p* ≤ 1 × 10^− 5^), with reductions of 3%, 6% and 9%, respectively.

For the bladder, when planning with the orig. PTV margins, the results indicated that the V_70Gy_ was approximately the same for both planning techniques, not showing any significant difference (*p* > 0.05). However, both the V_60Gy_ and V_50Gy_ were always smaller for the IMRT MRL plans (the reductions were, respectively, on average 0.7%, *p* = 0.019 and 1.1%, *p* = 0.002). For red. PTV margins, reductions ranging from 4% for V_70Gy_ to 6% for V_50Gy_ were observed with *p* ≤ 1 × 10^− 5^.

In terms of D_2%_ of the femoral heads, when planning with the orig. PTV margins, the IMRT MRL plans indicated on average a lower D_2 %_ for the left femoral head, with a reduction of nearly 2%, *p* = 0.001. Approximately the same D_2%_ values were found, for both techniques, for the right femoral head, with *p* > 0.05. When using the red. PTV margins, the D_2%_ was significantly lower in comparison to the VMAT plans (a reduction of nearly 6% for both left and right femoral heads), with *p* ≤ 1 × 10^− 5^.

The HI and CI are reported in Table 3, as well as the significance of the differences between the various modalities. The three modalities achieved a similar HI, with no statistically significant differences (*p* > 0.05). The CI were all < 1 and marginally higher for the VMAT plans than for both IMRT MRL plans, meaning the conformity achieved by VMAT’s dose distributions was slightly better, with *p* ≤ 0.049.

In Table 3 the distribution of the low dose is also summarised, where V_40Gy_ and V_25Gy_ are reported. Both parameters were larger for the IMRT MRL plans with orig. PTV margins, with a statistically significant difference for the V_25Gy_. As for the IMRT MRL plans with red. PTV margins, the V_40Gy_ and V_25Gy_ were both smaller compared to the VMAT plans, and the differences were both statistically significant.

Additionally, in Table 3 the plan parameters for treatment efficiency for the three types of plan presented in this study are shown. The number of MU for both IMRT MRL plans were significantly higher than for VMAT: an increase of 17% of the number of MU was reported for the IMRT MRL plans with orig. PTV margins, while for plans with red. PTV margins 25% more MU were needed. Treatment delivery times for IMRT MRL plans were calculated to be approximately 4.5 times longer than treatments delivered using VMAT plans on a conventional linac, which took on average 2.1 ± 0.0 min (*p* ≤ 1 × 10^− 5^).

**Table. 3:** Homogeneity and conformity indices for orig. VMAT and IMRT MRL plans with orig. PTV margins and red. PTV margins. Additionally, V_40Gy_ and V_25Gy_ and the average number of MU per fraction and the estimated treatment delivery time for the VMAT and IMRT MRL plans with orig. and red. PTV margins are shown. The results presented are mean values ± standard deviation. The *p*-values for the VMAT and IMRT MRL comparisons for both orig. and red. PTV margins are also indicated

	VMAT (orig. PTV margins)	IMRT MRL (orig. PTV margins)	IMRT MRL (red. PTV margins)
Homogeneity (HI)	0.08 ± 0.01	0.08 ± 0.01	0.08 ± 0.01
*p* value		0.7	0.4
Conformity (CI)	0.89 ± 0.05	0.87 ± 0.06	0.85 ± 0.05
*p* value		0.049	0.016
V_40Gy_ (% relative to VMAT)	100 ± 0.0	103 ± 8	81 ± 9
*p* value		0.1	≤ 1 × 10^− 5^
V_25Gy_ (% relative to VMAT)	100 ± 0.0	114 ± 9	89 ± 11
*p* value		≤ 1 × 10^− 5^	0.001
MU	639 ± 69	748 ± 61	796 ± 75
*p* value		≤ 1 × 10^− 5^	≤ 1 × 10^− 5^
Predicted treatment delivery time (min)	2.1 ± 0.0	9.4 ± 0.8	9.1 ± 0.6
*p* value		≤ 1 × 10^− 5^	1 × 10^− 5^

## Discussion

In the present planning study, VMAT and IMRT MRL plans with orig. PTV margins showed comparable dosimetric quality, with no clinically relevant differences regarding target coverage and dose to OARs. This is in agreement with the PCa results of Christiansen et al. and van de Schoot et al. for the high-field MR-linac [36, 39], where the same margins were used for both techniques and no clinically relevant differences regarding target coverage or doses to OARs were shown. Yadav et al. and Choi et al. compared low-field MR-linac-based IMRT plans to standard linac-based VMAT plans, for spine stereotactic body radiation therapy (SBRT), and both achieved comparable plan quality in terms of PTV DVH parameters [27, 30]. In our study, equivalent HI for both techniques were found, indicating a similar uniformity of the dose distribution in the target volume. However, better CI were observed for VMAT compared to IMRT MRL plans, corresponding to an increase of the irradiated volume for IMRT MRL orig. PTV margins. This is in contradiction to what Choi et al. observed for spine SBRT, where better CI were found for low-field MR-linac-based IMRT compared to VMAT plans. This disagreement is likely attributable to the different treatment sites, since our results are in agreement with Otto et al., who studied a novel-aperture based algorithm for VMAT treatment plan optimisation, and compared the resulting plan quality to a standard linac IMRT plan for nasopharyngeal carcinoma. In that study it was suggested that the limited number of angles in an IMRT technique may lead to poorer conformity [52]. Another study, from Palma et al., which investigated and compared 3D conformal radiotherapy with conventional linac IMRT and VMAT techniques for the treatment of PCa also proposed that the additional flexibility in dose delivery afforded by VMAT’s variable dose rate might contribute to a better dose conformity [25].

For the OARs, a similar sparing between IMRT MRL and VMAT plans was achieved. For the femoral heads, the IMRT MRL plans indicated a slightly lower, but significant, D_2%_ for the left femoral head in comparison to the VMAT plans (a reduction of about 2%), while the D_2%_ for the right femoral head was nearly the same for both techniques. Given that the patient structures are relatively symmetric, the difference between left and right femoral heads for orig. PTV margins was not expected. This difference was not seen when analysing the D_2%_ values for both femoral heads when red. PTV margins were used. This suggests that a larger sample size might be needed to determine whether this was a systematic effect.

Both V_40Gy_ and V_25Gy_ showed significant increases for the IMRT MRL plans. However, while the V_40Gy_ was only slightly higher in comparison to VMAT, the V_25Gy_ showed an increase of 14%. This follows the trend seen in Hoffmann et al., who evaluated VMAT and IMRT plans for PCa treatment delivery using a conventional linac. In this study the low dose bath of 40 Gy and 25 Gy isodose volumes, among other parameters, were analysed. Nearly the same V_40Gy_ values for IMRT and VMAT plans were reported, but a 62% increase for V_25Gy_ was observed [3].

With red. PTV margins, where the target volume was on average 1.3 times smaller than the orig. PTV, we observed similar DVH parameters for the target volume, while a reduction in the doses to the OARs was noticeable. Park et al. compared the plan quality of low-field MR-linac-based IMRT plans where the PTV volumes were approximately 4 times smaller, to conventional linac-based VMAT plans, for lung SBRT [28]. A better plan quality was attributed to the MR-linac-based IMRT technique, showing, in general, less dose to the surrounding normal tissue and a better target coverage [28]. Similarly to Park et al., the better outcomes for the OARs were attributed to the margin reduction capability, enabling the irradiation of smaller volumes [28]. This is also the explanation for the lower V_40Gy_ and V_25Gy_, determined for IMRT MRL plans with red. margins.

Several investigations have been made to find the most adequate margins to apply to the CTV for low and intermediate risk prostate cancer patients, considering the use of different types of IGRT and its frequency [53–55]. The choice for our orig. VMAT margins is based on the fact that our CT images have a slice thickness of 3 mm, on previous studies [56], and on our institutional practice and experience, with good tumour control and low toxicity observed among our patients. Naturally, the benefits of using the red. PTV margins for IMRT MRL plans would be less if we had used smaller orig. margins for the VMAT plans, or vice-versa.

For the IMRT MRL plans (with red. PTV margins), as mentioned before, the margin applied was slightly smaller in the superior-inferior direction than 4 mm due to the TPS’ features and the resolution of the CT used for treatment planning. Consequently, the dose to the OARs may have been slightly more reduced than with a 4 mm superior-inferior expansion, although it is not clear to what extent.

In terms of treatment delivery efficiency, the results suggested that the VMAT technique presents a higher efficiency in both scenarios, which concurs with other studies [27, 28, 30]. According to Choi et al., the average number of MU required for the low-field MR-linac-based IMRT plans was approximately 3 times larger than that for VMAT [27], whilst for Park et al. this number was 2 times larger [28]. In our study, this impact was less dramatic since this value was below 1.3 times for both plans, with orig. and with red. PTV margins.

However, it is also true that even when using the same delivery technique, the performance of different TPSs using different approaches can vary, leading to different outcomes. The performance of two commercial TPSs, Monaco (Elekta AB, Stockholm, Sweden) and Pinnacle (Philips Medical Systems, Madison, WI) for VMAT treatment plans regarding PCa was studied by Lafond et al. [57]. Both techniques offered clinically acceptable dose distributions with similar delivery times (on average 169 s for Monaco and 165 s for Pinnacle), despite the higher number of MU provided by Monaco, which needed 688 MU on average, as opposed to 452 MU for Pinnacle [57]. Moreover, Wiezorek et al. compared different rotational and static IMRT techniques for head-and-neck tumour treatment plans, where a comparable target coverage was achieved by all treatment plans [58]. However, discrepancies were found when analysing the treatment delivery times and the required number of MU. While the number of MU was, on average, slightly higher for the VMAT plans planned with Monaco (501 MU) in comparison to the VMAT plans planned with Eclipse (Varian, Palo Alto, USA) (437 MU), the shortest mean treatment times were associated with the latter, delivered in 2.5 min on average, in contrast to 9 min [58].

All three studies already mentioned, which compared low-field MR-linac-based IMRT to conventional linac-based VMAT plans, [27, 28, 30], reported beam-on times and not the estimated treatment delivery times. While Yadav et al. reported a beam-on time of approximately 2 times longer for MR-linac-based IMRT compared to VMAT, for Park et al. and for Choi et al. these values were, respectively, 4 times and 7 times larger. In our study, we decided to report the estimated treatment delivery time, since this represents the actual time needed for the treatment to be delivered, for which not only the beam-on time counts, but also the overhead to reposition the gantry and the time the MLC needs to form each segment are taken into consideration [26]. Treatment delivery times for both IMRT MRL plans were calculated to be below 4.5 times longer than treatments delivered using VMAT. The longer calculated treatment delivery times can be explained by various factors, such as the larger number of MU and the multiple field arrangement. This could raise concerns about a possible inaccurate delivery of the treatments due to patient and/or organ motion, with the consequence of dose delivery differences from that planned. However, the low-field MR-linac system has the ability to monitor the internal anatomy motion continuously and to deliver a gated beam, by the use of online cine MR images while the IMRT MRL plans are being delivered. This contributes for improved certainty of the position of the tumour and improved patient safety.

The reported differences between clinical VMAT and IMRT MRL plans (orig. PTV margins) were mainly due to differences in machine characteristics. Even though the exact contribution of each factor could not be identified in this study, discrepancies were mostly induced by treatment machine geometries (MLC leaf width, collimator positioning, and dose rate), differing TPSs, treatment techniques and the presence of the magnetic field.

It is important to mention that such studies carry the risk of bias, especially when clinical plans, with a well-established technique, are compared to plans created using a novel technique. Another limiting factor could be the fact that VMAT and IMRT MRL plans were produced by different planners. Nevertheless, the results were consistent and all the IMRT MRL plans were considered clinically acceptable and equivalent to the clinical ones. To evaluate whether performing the DVH analysis of all plans on the same TPS used to generate the IMRT MRL plans introduced a bias in our study, an independent system, Oncentra MasterPlan (Nucletron B.V., Veenendaal, Netherlands), was used for repeated DVH analysis for 5 out of the 20 patients. The DVH parameters for the target volume and the OARs were compared to the ones reported by the MRIdian system and the resulting deviations between both TPSs were always below 0.5%, demonstrating a good agreement.

## Conclusions

This study demonstrated that, under similar conditions, the MRIdian system is capable of delivering PCa treatment plans of similar dosimetric quality as conventional linac-based VMAT plans. Furthermore, a better sparing of the surrounding OARs and healthy tissue is possible due to the margin reduction enabled by online MR image guidance.

## Data Availability

The datasets generated and/or analysed during the current study are not publicly available due to patient privacy concerns.

## Abbreviations

CBCT: cone beam computed tomography
CI: conformity index
CT: computed tomography
CTV: clinical target volume
DVH: dose-volume histogram
EBRT: external beam radiation therapy
FFF: flattening-filter-free
HI: homogeneity index
IGRT: image guided radiation therapy
IMRT: Intensity modulated radiation therapy
linac: linear accelerator
MLC: multileaf collimator
MR: magnetic resonance
MRgRT: MR-guided RT
MU: monitor units
OAR: organ at risk
PCa: prostate cancer
SBRT: stereotactic body radiation therapy
ssIMRT: step-and-shoot IMRT
TPS: treatment planning system
US: ultrasound
VMAT: volumetric modulated arc therapy
